# Healthacross initiative of Lower Austria

**DOI:** 10.1093/eurpub/ckac129.486

**Published:** 2022-10-25

**Authors:** J Winkler

**Affiliations:** Healthacross Initiative, Health Agency of Lower Austria, St. Pölten, Austria

## Abstract

Lower Austria is by size the biggest province of Austria and located in the heart of Europe with a 414 km long border to the Czech Republic and Slovakia, and in close proximity to Hungary. Not only through its geographic location but also through its understanding to be a connected region as part of a strong European Union, cross-border health care plays an important issue at the provincial level. Led by the European spirit, all international and cross-border health activities are bundled under the umbrella of the ‘Healthacross initiative”. The initiative is part of the Health Agency of Lower Austria (N&Ouml; Landesgesundheitsagentur), which operates and manages all 27 public hospitals and around 50 care/nursing homes in the region. Via the “Healthacross initiative”, Lower Austria is participating in four transnational networks and since 2008, conducted eight EU co-funded projects in cross-border healthcare, four of them currently running. Healthacross is the coordinator of this session, with support of WHO RHN network. Active networking in various European and international networks is part of the regional strategy of Lower Austria in order to gain excellence to improve the health and quality of life for citizens living in border regions. The premise is to enable equal access to health care services for citizens, regardless of their place of residence. The current epidemiological situation with the COVID-19 pandemic has not only impact on cross-border healthcare, but also on the life, health and well-being of citizens. With the closure of national borders, the exchange of healthcare services came to an interim halt; solely dialog remained - which was incredibly valuable for both commuters and citizens living in the border regions. Lower Austria will take care of the moderation of the session and will be represented by Julia Winkler.

